# Autonomic Nervous System: From Bench to Bedside

**DOI:** 10.3390/jcm9103180

**Published:** 2020-09-30

**Authors:** Eleonora Tobaldini

**Affiliations:** 1 Department of Internal Medicine, Fondazione IRCCS Ca’ Granda, Ospedale Maggiore Policlinico, 20122 Milan, Italy; eleonora.tobaldini@unimi.it; 2 Department of Clinical Sciences and Community Health, University of Milan, 20122 Milan, Italy

In recent decades, new evidence has suggested that the role of the Autonomic Nervous System (ANS) is not marginal and not only limited to controlling vegetative functions. The ANS is a complex system, crucial for the onset and the progression of cardiovascular but also non-cardiovascular disorders and it is highly integrated with other biological pathways, from inflammation to the immune system. Therefore, the possibility to target the ANS in pathological conditions has become a new promising therapeutic approach in several non-communicable diseases.

To cover these important topics, this Special Issue has been dedicated to integrative approaches for the assessment of ANS, both in animal and human experimental settings, covering different areas of expertise.

Starting from the discussion of important technical aspects, Alcantara and colleagues suggested that the application of different Kubios filters could strongly impact on Heart Rate Variability (HRV) parameters and must be kept in mind when performing time and frequency-domain analysis [1].

The status of the ANS is an independent prognostic factor for adverse cardiovascular outcomes and the ANS has been recognized as a major determinant of health and prognosis, as reported by two interesting studies. In an animal model, Carnevali and colleagues, suggested that in populations without known cardiac disease, resting HRV can predict ventricular arrhythmias, especially in conditions of strong sympathetic activation [2]. A second study by Jarczok et al. showed that a single value of Root Mean Square of the Successive Differences (RMSSD), an index derived from time-domain analysis of HRV, can be linked to a higher risk across a range of well-known cardiovascular risk factors, being a possible easy method to assess novel marker of cardiovascular risk [3]. 

Traditionally, the role of ANS has been widely investigated in cardiovascular disorders. On this topic, an original article investigated the role of sympathetic baroreflex control during orthostatic pre-syncope, suggesting that a negligible baroreceptor modulation in this setting might alter the sympathetic discharge pattern, impairing the capability of vessels to constrict and thus, leading to pre-syncope [4].

Novel aspects of neural autonomic control are emerging from studies dealing with non-cardiovascular disorders, such as neurological and pulmonary diseases. In fact, three papers explored the role of ANS in acute and chronic neurological disorders, such as Restless Legs Syndrome/Periodic Limb Movements, stroke, and multiple sclerosis. The study by Sforza and colleagues showed that a significant increase in sympathetic activity occurred in periods with periodic limb movements and electromyogram power is the factor that affects sympathetic control [5]. Lin et al. observed that baroreflex sensitivity can predict functional outcomes and complications after acute ischemic stroke [6]. Finally, a very interesting systematic review on cardiac autonomic dysfunction in multiple sclerosis by Findling et al. reported that no studies have yet looked at whether treating cardiac autonomic dysfunction can reduce the burden of multiple sclerosis symptoms, disease activity, or the rate of progression, suggesting that future studies are required to elucidate the role of the ANS in multiple sclerosis [7]. Similarly, the application of HRV in other settings such as pulmonary diseases revealed interesting novel data. In fact, in comparison to healthy subjects, patients with end-stage lung diseases awaiting lung transplantation showed a predominant sympathetic modulation and lower markers of vagal modulation, but the more severe the disease, the lower the sympathetic control [8].

All together, these papers provide evidence that the ANS plays a pivotal role in the pathogenesis and in the progression of non-cardiovascular disorders.

The impact of the ANS must be considered also when discussing about female health. A strong link between menstrual cycle and ANS modifications has been reported by two papers by Schmalenberger et al. In the original article on the relation between HRV and menstrual hormones [9], the authors showed that progesterone is correlated with HRV, while higher-than-usual progesterone levels predicted lower HRV. The mid-luteal phase was characterized by lower levels of HRV, while no effects were found when considering estradiol. Together with their meta-analysis [10] that strongly indicated the presence of vagal modifications across the menstrual cycle, these two studies underline the importance of considering cycle phases when assessing HRV in women.

Physiological conditions, such as physical exercise and ageing, are strongly interconnected with the ANS. As for physical exercise, two papers explore the link between physical exercise and cardiovascular autonomic control. In detail, the study by Gronwald assessed the effects on the ANS of short-term cycling interval sessions and active recovery in endurance trained cyclists, applying nonlinear dynamics for the analysis of the ANS [11]. On the other hand, Vitale et al. explored the role of the time of day and chronotype HRV in sport performances, suggesting that the control for chronotype and time-of-day effect can play an important role for individualized training schedules and possibly primary injury prevention in athletes [12].

As for the genetic factors affecting the ANS, a genome-wide association study evaluated the possible role of two specific genetic polymorphisms as markers for cognitive impairment in hypotension [13], while Chien-Hwa Wu addressed the issue of the relationship between cardiac autonomic control and nutritional status in patients undergoing chronic hemodialysis. They suggested that an altered ANS function could link malnutrition to worse prognosis in hemodialysis patients [14].

Two review articles, one on sleep apnea, hypertension, and the sympathetic nervous system [15] and the other one on the effects of energy drinks on cardiovascular autonomic responses, explored interesting topics with quite broad clinical impacts. In the first one, the authors underlined and updated the important link between sleep apnea, the most common sleep-disordered breathing (SDB), hypertension, and the ANS. Sleep apnea is very common in patients affected by cardiovascular disorders, and the comorbidity with hypertension has great clinical relevance. In this setting, the alterations of the ANS, characterized by sympathetic overactivity, play a key role in linking SDB to increased cardiovascular morbidity and mortality. The narrative review by Somers and Svatikova [16] tried to shed new light on the relation between energy drinks and cardiovascular autonomic control. The study reported that energy drinks may induce adverse cardiovascular events (such as atrial fibrillation, ventricular arrhythmia, myocardial infarction, and sudden death), even in otherwise healthy subjects and that the ANS could play a pivotal role in this condition.

Finally, the role of the ANS as a possible therapeutic target for cardiovascular disorders is gaining a lot of interest from clinicians. In this perspective, two studies on the possibility to target the ANS in a non-pharmacological way have been published. The paper by Kiuchi and colleagues explored the topic of renal denervation to treat hypertension in a cohort of patients with chronic kidney disease. This study found a significant correlation between the number of ablated sites in renal arteries and the systolic blood pressure lowering effect in the long-term, thus underlining the real positive effect of renal ablation to treat hypertension [17]. On the other side, a study conducted in healthy subjects on the effects of transcutaneous vagus nerve stimulation (tVNS) showed that short term tVNS lowered heart rate and affected cardiac and peripheral autonomic control at rest and during orthostatic stimuli, thus suggesting that tVNS could be, in the future, a useful and effective method to target ANS in several different pathological conditions [18].

In conclusion, ANS can be considered a highly integrated system, strongly interconnected with other biological systems such as inflammation, hormonal regulation, immune system, and others, modulated by physiological conditions such as exercise and sleep and with a great impact in cardiovascular and non-cardiovascular diseases.

The content of this Special Issue is aimed at shedding new light on the complex regulation and integration of ANS both in health and disease.
